# Behçet's disease with intestinal involvement: a case report and review of the literature

**DOI:** 10.1186/s13256-023-04148-w

**Published:** 2023-10-06

**Authors:** Lin Li, Jing Wang, Huifang Li, Chiyi He, Xiaoping Niu

**Affiliations:** 1 Departments of Gastroenterology, The First Affiliated Hospital of Wannan Medical College, Wuhu, 241001 People’s Republic of China; 2 Departments of Gastroenterology, Yijishan Hospital of Wannan Medical College, Wuhu, 241001 People’s Republic of China

**Keywords:** Behçet's disease, Intestinal ulcer, Vasculitis, Adalimumab

## Abstract

**Background:**

Behçet’s disease (BD) is a chronic systemic disease characterized by vasculitis as the basic pathological change. BD is rare, and gastrointestinal involvement occurs in 3% to 25% of affected patients. This article describes a rare case of intestinal BD along with a literature review of intestinal involvement in BD.

**Case presentation:**

A 50-year-old Han woman from China presented with a > 6-month history of distending pain in the right upper abdomen. Because of mechanical obstruction secondary to stricture formation from an ileocecal ulcer, she underwent radical right colon resection, and postoperative pathologic examination indicated an ileocecal ulcer. The patient was readmitted to the hospital 6 months postoperatively for recurrence of the same symptoms. Colonoscopy indicated obvious narrowing of the anastomosis with an oval-shaped deep ulcer that could not be passed by the endoscope. Pathologic examination showed acute and chronic inflammation of the anastomotic mucosa and granulation tissue. In addition, gastroscopy showed a 3.0- × 4.0-cm giant ulcer at the junction of the descending bulb along with a sinus tract. Moreover, total gastrointestinal computed tomography angiography showed significant thickening of the intestinal wall near the transverse colon, forming a sinus tract at the junction of the antrum and duodenum with a length of about 1.3 cm and width of about 0.2 cm. Further inquiry regarding the patient’s medical history revealed that she had developed repeated oral ulcers 3 years previously and repeated eye inflammation 5 years previously. Specimens of the right half of the colon removed 6 months previously were sent to Run Run Shaw Hospital Affiliated to Zhejiang University for consultation. The pathologic examination revealed vasculitis in the submucosa and subserosa, and the patient was finally diagnosed with BD. She began treatment with adalimumab, and repeat gastroenteroscopy revealed that the intestinal ulcer had significantly improved.

**Conclusions:**

An oval-shaped deep intestinal ulcer is a characteristic lesion in patients with BD and may involve the intestinal muscle layer. This case emphasizes that BD is a vasculitis affecting multiple organs and can present with a single, deep, clean-edged intestinal ulcer that penetrates the bowel wall to form a sinus tract. Therefore, careful examination and differential diagnosis should be carried out to prevent a poor prognosis. Adalimumab is effective for patients with intestinal BD.

## Background

Behçet’s disease (BD) was first reported and confirmed by Behçet in 1937 [1]. It is a disease of unknown etiology and is characterized by small vessel vasculitis as the pathological basis. BD mainly involves the skin and mucous membranes, and it induces chronic progressive damage to multiple body systems over time [2, 3].

The clinical manifestations of BD vary, and there are no specific laboratory biomarkers that support a definitive diagnosis. The diagnosis of BD is mainly based on clinical manifestations; this makes it difficult to identify BD at its onset, leading to delayed diagnosis [4]. The pathogenesis of BD is unknown. Pathological assessment is the main diagnostic method and usually shows vasculitis.

BD is rare and is characterized not only by recurrent oral and genital aphthous ulceration but also cutaneous, ocular, vascular, and/or gastrointestinal lesions [5]. Gastrointestinal involvement occurs in 3% to 25% of patients with BD, and the incidence of gastrointestinal involvement is higher in East Asian countries such as Korea and Japan than in Western or Middle Eastern countries [6]. The gastrointestinal manifestations of BD usually occur in the ileocecal region and are characterized by right lower abdominal pain, abdominal distension, diarrhea, and hematochezia. Thus, BD is easily confused with inflammatory bowel disease.

The purpose of this report is to describe a rare case of BD with intestinal involvement that was eventually confirmed by clinical and pathological findings. Clinical differential diagnosis and pathological findings are particularly important in the diagnosis of BD.

## Case presentation

On 1 November 2020, a 50-year-old Han woman presented with a 6-month history of recurrent distending pain in the right upper abdomen and was admitted to the Department of Gastrointestinal Surgery at The First Affiliated Hospital of Wannan Medical College. The abdominal pain was paroxysmal and accompanied by nausea and vomiting. She had no diarrhea, no black or bloody stools, and no fever or chills. Physical examination revealed tenderness and rebound pain in the middle and upper abdomen, no muscle tension, no palpable abdominal mass, no signs of oral or vulvar ulcers, no perianal fistula, and no joint rash.

Laboratory investigations revealed normocytic anemia (hemoglobin, 97 g/L), hypoproteinemia (albumin, 29.6 g/L), and elevated inflammatory marker levels [C-reactive protein (CRP), 10.47 mg/L; erythrocyte sedimentation rate (ESR), 28 mm/h]. The platelet count was 305 × 10^9^/L, exceeding the upper limit of normal. Positive results were obtained for fecal occult blood, tuberculosis antibody, tuberculosis infection based on the number of spot-forming T cells (T-SPOT.TB test), and tuberculin purified protein derivative. No obvious abnormality was observed in the white cell count, blood coagulation, routine laboratory evaluation before blood transfusion, tumor markers, and an acid-fast bacillus sputum smear.

**Fig. 1:**
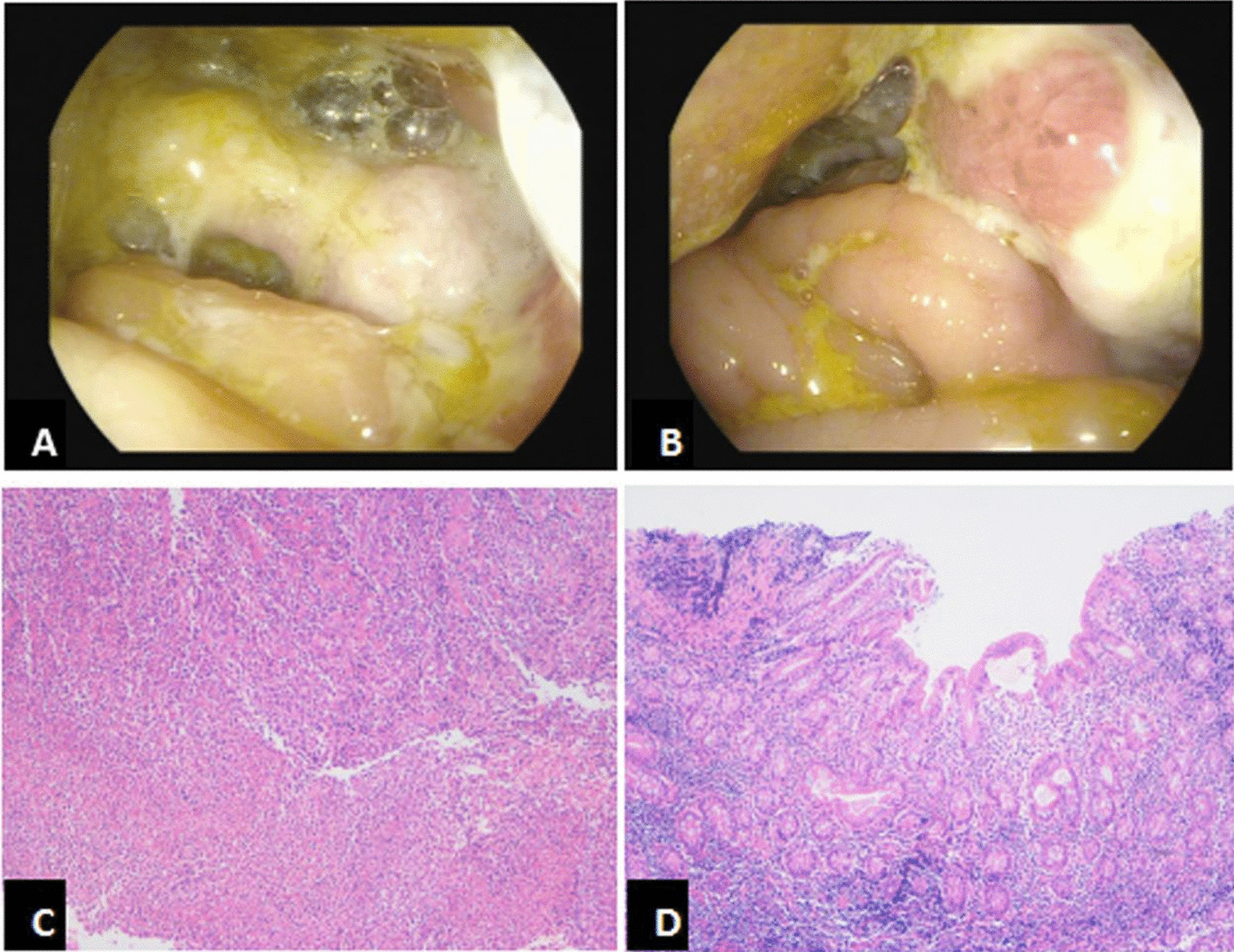
**A**, **B** Colonoscopy showed a large circumferential ulcer infiltrating from the proximal ascending colon to the ileocecal part, and the intestinal cavity was obviously narrow; **C**, **D** pathology showed that acute and chronic inflammation of the mucosa, with crypt abscess, inflammatory exudation, necrosis and granulation tissue, ulcer, but no granulomatous inflammation

Because of the patient’s normocytic anemia and right upper abdominal pain, enhanced computed tomography (CT) of the whole abdomen was carried out first. The results indicated obvious thickening and strengthening of the cecum and ascending colon with multiple enlarged lymph nodes, raising the possibility of cancer. Colonoscopy subsequently showed a large circumferential ulcer infiltrating the bowel from the proximal ascending colon to the ileocecal region, and the intestinal cavity was too narrow for passage of the endoscope (Fig. 1A, B). Pathologic examination showed acute and chronic inflammation of the mucosa with crypt abscesses, inflammatory exudation, necrosis, and granulation tissue, but no granulomatous inflammation (Fig. 1C, D). Because of the mechanical obstruction secondary to the stricture from the ileocecal ulcer, part of the intestine in the right abdomen was dilated with gas and fluid accumulation. After failure of conservative treatment (fasting, gastrointestinal decompression, and anti-infection agents), the patient finally underwent radical right colon resection on 4 November 2020. Postoperative pathologic examination revealed a 7.0- × 4.0-cm ileocecal ulcer. The peri-intestinal wall tissue was infiltrated by numerous acute and chronic inflammatory cells and contained lymphatic follicles. Both the peri-ileal and pericolic lymph nodes showed reactive hyperplasia.

On 10 December 2020 (1 month after the operation), the patient was readmitted to the hospital because of abdominal pain. Abdominal CT revealed intestinal obstruction and pelvic effusion. After symptomatic treatment consisting of fasting, fluid rehydration, and anti-infection treatment, the patient was discharged with improved symptoms.

**Fig. 2:**
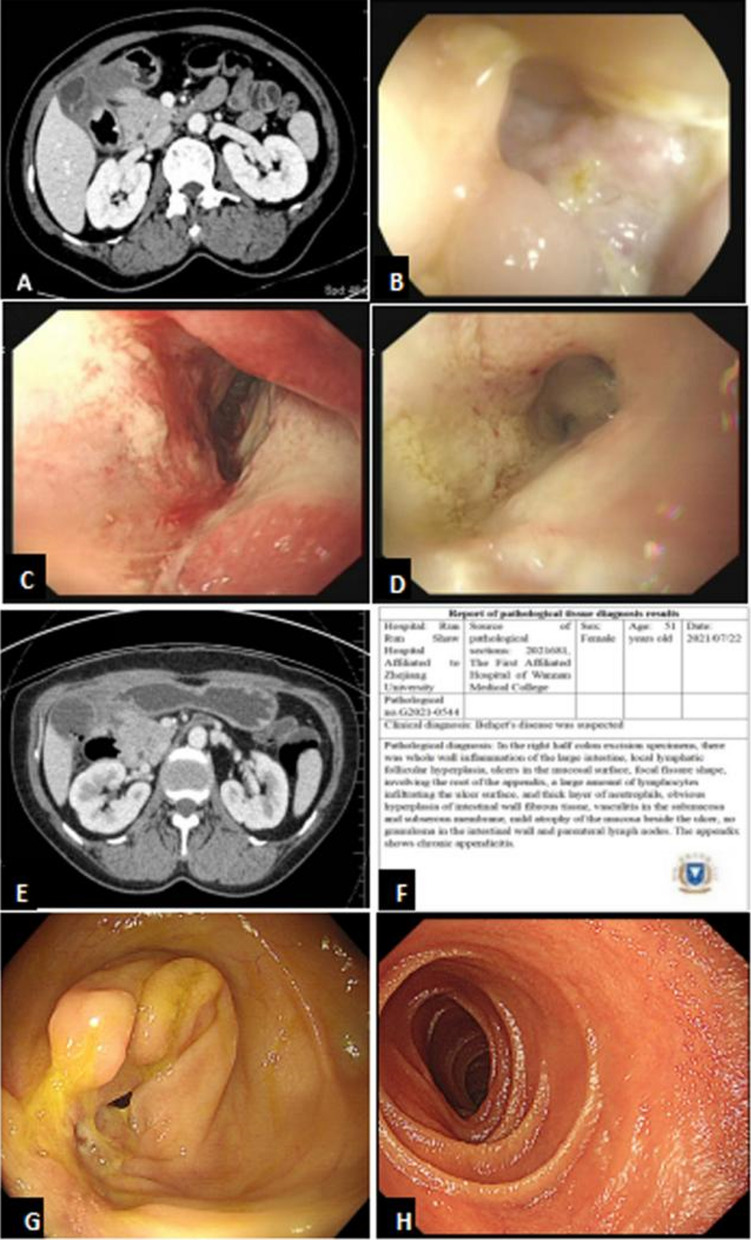
**A** Abdominal enhanced CT showed a little exudation in the fatty space around the anastomosis and multiple lymph nodes, uneven thickening and edema of the gastric wall in the gastric pylorus; **B** colonoscopy showed that the anastomosis was obviously narrow with huge ulcers, which could not be passed by the endoscope; **C**, **D** gastroscopy showed that a 3.0 cm*4.0 cm giant ulcer was observed at the junction of descending bulb and the sinus tract was formed; **E** a total gastrointestinal CT angiography showed that significant thickening of the intestinal wall near the transverse colon, forming a sinus tract at the junction of the sinus-duodenum with a length of about 1.3 cm and a width of about 0.2 cm; **F** pathology showed that vasculitis could be seen in the submucosa and subserosa, and the patient was diagnosed as BD; **G**, **H** repeat gastroenteroscopy revealed that the intestinal ulcer was significantly improved

Six months later, the patient presented to the Department of Gastroenterology because of a 5-day history of distending pain in the right upper abdomen. Physical examination showed no abdominal tenderness or rebound pain, no palpable abdominal mass, no bulbar conjunctival congestion, no oral or vulvar ulcers, no perianal fistula, and no joint rash or other abnormalities. A plain CT scan indicated irregular thickening of the intestinal wall in the operative area, which seemed to be locally connected to the descending part of the duodenum. Laboratory investigations revealed normocytic anemia (hemoglobin, 100 g/L), hypoproteinemia (albumin, 33.7 g/L), elevated inflammatory marker levels (CRP, 13.77 mg/L; ESR, 50 mm/h), and a high platelet count of 320 × 10^9^/L. Tuberculosis antibody, the T-SPOT.TB test, and tuberculin purified protein derivative were positive. However, negative results were obtained on an acid-fast bacillus sputum smear, fecal occult blood test, blood coagulation profile, tumor marker measurement, autoantibody measurement, and antineutrophil cytoplasmic antibody measurement. Abdominal enhanced CT showed a small amount of exudation in the fatty space around the anastomosis and multiple lymph nodes as well as uneven thickening and edema of the wall of the gastric pylorus (Fig. 2A). Colonoscopy showed obvious narrowing of the anastomosis with a huge deep ulcer that could not be passed by the endoscope; no abnormalities were found in the remaining intestinal tract (Fig. 2B). Pathologic examination showed acute and chronic inflammation of the anastomotic mucosa, inflammatory exudation, necrosis, and granulation tissue. In addition, gastroscopy showed a 3.0- × 4.0-cm giant ulcer at the junction of the descending bulb. The ulcer was covered with yellow slough, and a sinus tract had formed (Fig. 2C, D). Moreover, total gastrointestinal CT angiography showed significant thickening of the intestinal wall near the transverse colon, forming a sinus tract at the junction of the antrum and duodenum with a length of about 1.3 cm and width of about 0.2 cm (Fig. 2E). Further inquiry regarding the patient’s medical history indicated that she had experienced repeated oral ulcers 3 years previously and repeated eye inflammation 5 years previously, neither of which had recurred since then. Based on the above results, specimens of the right half of the colon removed 6 months previously were sent to Run Run Shaw Hospital Affiliated to Zhejiang University for consultation. The pathologic examination revealed vasculitis in the submucosa and subserosa, and the patient was diagnosed with BD (Fig. 1F). Thus, we ultimately diagnosed BD according to the International Criteria for Behçet’s Disease (ICBD) [7].

The patient was treated with methylprednisolone (40 mg) and isoniazid (0.4 g) four times daily for 3 weeks, followed by a tapering of prednisone and adalimumab 2 weeks once for 6 months. This resulted in resolution of the patient’s abdominal distension as well as weight gain. Repeat gastroenteroscopy revealed that the intestinal ulcer had significantly improved (Fig. 2G–I). Repeat laboratory investigations after 6 months of treatment showed the following: hemoglobin, 132 g/L; platelet count, 253 × 10^9^/L; albumin, 44.3 g/L; CRP, 0.88 mg/L; and ESR, 4.7 mm/h.

## Discussion and conclusions

This case report describes a middle-aged woman with a chronic disease course. At the first visit, because of the patient’s atypical clinical manifestations and aggravation of intestinal obstruction, radical right colon resection was performed, and postoperative pathologic examination indicated ulcerative lesions. The patient was readmitted to the hospital 6 months postoperatively for recurrence of the same symptoms. After a series of examinations, follow-up of her medical history (repeated oral ulcers and eye inflammation), and consideration of pathological findings indicating vasculitis, the patient was finally diagnosed with BD.

In 1990, the International Group of BD first defined the diagnostic criteria for BD: recurrent oral ulcers plus any two of the following: recurrent genital ulcers, eye lesions, skin lesions, and positive acupuncture tests. In 2014, new diagnostic criteria for BD (the ICBD) were proposed, including two additional clinical criteria: neurological disease and vascular involvement. Notably, these allow for diagnosis of BD even in the absence of oral ulcers. However, these diagnostic criteria do not include intestinal symptoms and signs [7].

We used the ICBD to verify the diagnosis in the present case. Ocular lesions, oral lesions, and genital lesions are assigned 2 points each, while skin lesions, central nervous involvement, vascular manifestations, and systemic involvement are assigned 1 point each. Positive pathological tests are assigned 1 point. Patients who score ≥ 4 points are classified as having BD. Our patient scored 5 points using this system: ocular lesions (2 points), oral lesions (2 points), and pathological tests (1 point). Therefore, she was finally diagnosed with BD.

Intestinal BD is a type of vasculitis that usually occurs in the ileocecal region. It manifests as ulcer-like changes in the damaged intestinal mucosa. The ulcers are deep, large, and perforated. BD is a multisystem disease that can often involve blood vessels, nerves, and joints; however, it rarely occurs in the digestive system. Increasingly more studies on gastrointestinal BD have been performed in recent years, but the rates of missed diagnosis and misdiagnosis are high because of its complex and diverse clinical manifestations and lack of specificity. Numerous studies have shown that abdominal pain is the most common clinical manifestation of intestinal BD, which is consistent with the present case. Some patients also have black or bloody stool, digestive tract perforation, or a perianal abscess as the first symptom [8–10]. Although the pathogenesis of BD remains unknown, it may be caused by genetic, immune, infective, or environmental factors.

Intestinal BD generally appears 4 to 6 years after oral ulcers. In the present case, the intestinal ulcer appeared 3 years after the oral ulcer and 5 years after the eye disease. Neither the oral ulcer nor eye disease recurred after cure was attained, which increased the difficulty of diagnosis in this case. Therefore, it is particularly important to obtain a BD-related medical history of patients with intestinal ulcers, especially those with a single ulcer.

An oval-shaped deep intestinal ulcer is a characteristic lesion in patients with BD, and such an ulcer may involve the intestinal muscle layer. BD-related ulcers usually occur as a solitary lesion, and their site of onset is usually in the terminal ileum and ileocecal region; they are not commonly seen in the upper digestive tract. Thus, a possible complication of intestinal BD is intestinal perforation, especially ileocecal perforation. One report described a case of spontaneous ileocecal perforation in a patient with BD, and life-threatening iatrogenic bowel perforation occurred during colonoscopy [11]. In the present case, abdominal CT and total gastrointestinal CT angiography showed the formation of a duodenal sinus between the anastomosis and the duodenal bulb. Based on the above evidence, we assumed that the duodenal ulcer was secondary to the anastomotic ulcer because sinus was present between the duodenum and anastomosis. The deep, large duodenal ulcer and the formation of the duodenal sinus were caused by the ulcer outside the anastomosis infiltrating the intestinal muscle layer. Like Crohn’s disease (CD), BD can also cause the formation of a penetrating sinus in the digestive tract. Clinicians should be alert to the rare complications of BD.

BD is characterized by oral ulcers, pigmented membrane inflammation, and skin injury, whereas CD is mainly characterized by extensive intestinal injury and granulomatous enteritis [6]. It is difficult to distinguish intestinal ulcers caused by BD from other inflammatory intestinal lesions, especially those caused by CD, which shares common extra-enteral manifestations such as ocular uveitis, arthritis, oral ulcers, pyoderma gangrenosum, vascular occlusive disease, and thrombosis. Gastrointestinal involvement in BD is similar to that in CD, but there are still differences. Deep round ulcers with clear edges in the ileocecal region suggest BD [12]. However, segmental longitudinal ulcers and a pebble-like appearance are more likely to indicate CD. Gastrointestinal biopsy specimens are the most common tissues used for pathological assessment. Obvious vasculitis is the most common feature of BD [12, 13]. Therefore, a detailed history, clinical features, and pathological assessment have important diagnostic value.

At present, there is no recognized radical treatment of intestinal BS. Glucocorticoids and immunosuppressants are commonly used to improve the patient’s clinical condition according to the European Alliance of Associations for Rheumatology guidelines [5]. However, monoclonal anti-tumor necrosis factor antibodies (infliximab or adalimumab) can be considered for patients with refractory intestinal BD [14–16]. One case report revealed that infliximab-induced remission of intestinal BD was mediated not only by inhibiting the tumor necrosis factor-α–mediated signaling pathway but also by promoting interleukin-6 expression and accumulation of regulatory T cells expressing FOXP3 [17]. Zhao *et al.* [18] recently found that tofacitinib (a Janus kinase inhibitor) is effective against refractory intestinal BD and that Janus kinase inhibitors can be potential therapeutic targets for the treatment of severe refractory intestinal BD [18]. Surgery should be performed for patients with serious complications such as severe gastrointestinal bleeding, perforation, fistulas, intestinal obstruction, and huge abdominal masses. In the present case, the symptoms of intestinal obstruction were aggravated and the patient underwent surgical treatment. After readmission, she was finally diagnosed with intestinal BD. After treatment with adalimumab, the patient’s symptoms were relieved and the ulcer healed. Adalimumab has been successfully used as a first-line anti-TNF-α agent in steroid-dependent intestinal BD patients to induce and maintain complete response [19].

Overall, the prognosis of this patient was good. BD should be considered in patients with ileocecal ulcerative lesions, especially single ulcers; recurrent abdominal pain; and parenteral symptoms other than those caused by inflammatory bowel disease. The diagnosis of BD relies upon a combination of clinical, radiological, endoscopic, and histological findings. The typical pathological features of BD are difficult to identify, and the experience of the pathologist will also affect the determination of the final outcome, which is a limitation in our study. Establishing a precise diagnosis can be difficult, but it is important because the treatment and prognosis can be highly variable.

In conclusion, intestinal involvement in BD is rare in clinical practice. The diagnosis of BD is challenging because of its atypical symptoms. The clinical cases described in this report highlight the importance of identifying intestinal involvement in BD and the difficulty of diagnosing such patients. The additional examination methods are nonspecific, and the patient’s clinical presentation, medical history, and pathological findings provide the basis for diagnosis. This case emphasizes that BD is a vasculitis that affects multiple organs and can present with a single clean-edged ulcer that penetrates to form a sinus tract in the intestine. Therefore, careful examination and differential diagnosis are necessary. Adalimumab is effective for patients with intestinal BD. Future studies are needed for the development of new diagnostic criteria for BD that include different clinical manifestations, such as gastrointestinal involvement.

## Data Availability

All information about the patient come from departments of Gastroenterology Surgery, Gastroenterology and Pathology, Yijishan Hospital of Wannan Medical College. The data underlying this article are available in the article and will be shared on reasonable request to the corresponding author. CARE Checklist of information is included in this case-report manuscript.

## Abbreviations

BD: Behçet’s disease
CRP: C-reactive protein
ESR: Erythrocyte sedimentation rate
CT: Computed tomography
ICBD: International Criteria for Behçet’s Disease
CD: Crohn’s disease

## References

[1] Behçet H (1937). Rezidivierende aphthose, durch ein virus verusachte geschwure am auge und an den genitalien. Dermatol Wochenschr.

[2] Saleh Z, Arayssi T (2014). Update on the therapy of Behçet disease. Ther Adv Chronic Dis.

[3] Hamdan A, Mansour W, Uthman I, Masri AF, Nasr F, Arayssi T (2006). Behçet’s disease in Lebanon: clinical profile, severity and two-decade comparison. Clin Rheumatol.

[4] Davatchi F, Chams-Davatchi C, Shams H (2017). Behcet’s disease: epidemiology, clinical manifestations, and diagnosis. Expert Rev Clin Immunol.

[5] Hatemi G, Christensen R, Bang D, Bodaghi B, Celik AF, Fortune F (2018). 2018 update of the EULAR recommendations for the management of Behçet’s syndrome. Ann Rheum Dis.

[6] Ananthakrishnan AN (2015). Epidemiology and risk factors for IBD. Nat Rev Gastroenterol Hepatol.

[7] Davatchi F, Assaad-Khalil S, Calamia KT, Crook JE, Sadeghi-Abdollahi B, Schirmer M, Tzellos T, Zouboulis CC, Akhlagi M, Al-Dalaan A, Alekberova ZS, Ali AA, Altenburg A, Arromdee E, Baltaci M, Bastos M, Benamour S, Ben Ghorbel I, Boyvat A, Carvalho L, Chen W, Ben-Chetrit E, Chams-Davatchi C, Correia JA, Crespo J, Dias C, Dong Y, Paixão-Duarte F, Elmuntaser K, Elonakov AV, Graña Gil J, Haghdoost A-A, Hayani RM, Houman H, Isayeva AR, Jamshidi AR, Kaklamanis P, Kumar A, Kyrgidis A, Madanat W, Nadji A, Namba K, Ohno S, Olivieri I, Vaz Patto J, Pipitone N, de Queiroz MV, Ramos F, Resende C, Rosa CM, Salvarani C, Serra MJ, Shahram F, Shams H, Sharquie KE, Sliti-Khanfir M, Tribolet de Abreu T, Vasconcelos C, Vedes J, Wechsler B, Cheng YK, Zhang Z, Ziaei N (2014). The International Criteria for Behcet's Disease (ICBD): a study of 27 countries on the sensitivity and specificity of the new criteria. J Eur Acad Dermatol Venereol.

[8] Fujita H, Kiriyama M, Kawamura T, Ii T, Takegawa S, Dohba S, Kojima Y, Adachi H, Morimoto H, Kobayashi A, Watanabe K (2002). Massive hemorrhage in a patient with intestinal Behcet’s disease: report of a case. Surg Today.

[9] Turan M, Sen M, Koyuncu A, Aydin C, Aricis S (2003). Sigmoid colon perforation as an unusual complication of Behcet’s syndrome: report of a case. Surg Today.

[10] Haller C, Guenot C, Odman M, Bruttin JM, Rosso R (2003). Recurrent anal abscess and cecal perforation as a first presentation of Behcet’s disease. Gastroenterol Clin Biol.

[11] Mu T, Feng H (2022). Bilateral pneumothorax and pneumomediastinum during colonoscopy in a patient with intestinal Behcet's disease: a case report. World J Clin Cases.

[12] Geboes K, Dalle I (2002). Vasculitis and the gastrointestinal tract. Acta Gastroenterol Belg.

[13] Dzhus MB, Karasevska TA, Tsaralunga VM, Yurchenko AV, Ivashkivsky OI (2022). Behçet's disease with intestinal involvement: case-based review. Rheumatol Int.

[14] Jennette JC, Falk RJ, Bacon PA, Basu N, Cid MC, Ferrario F (2012). Revised international chapel hill consensus conference nomenclature of vasculitides. Arthr Rheum.

[15] Lee JH, Cheon JH, Jeon SW, Ye BD, Yang SK, Kim YH (2013). Efficacy of infliximab in intestinal behçet’s disease: a Korean multicenter retrospective study. Inflamm Bowel Dis.

[16] Suzuki Y, Hagiwara T, Kobayashi M, Morita K, Shimamoto T, Hibi T (2021). Long-term safety and effectiveness of adalimumab in 462 patients with intestinal Behçet’s disease: results from a large real-world observational study. Intest Res.

[17] Yoshikawa K, Watanabe T, Sekai I (2021). Case report: a case of intestinal Behçet's disease exhibiting enhanced expression of IL-6 and Forkhead Box P3 mRNA after treatment with infliximab. Front Med (Lausanne).

[18] Zhao N, Tang Y, Wang S, Cui L, Sun X, Wang Z, Liu Y (2022). Case report: refractory intestinal Behçet's syndrome successfully treated with tofacitinib: a report of four cases. Front Immunol.

[19] De Cassan C, De Vroey B, Dussault C (2011). Successful treatment with adalimumab in a familial case of gastrointestinal Behçet’s disease. J Crohns Colitis.

